# An Essential Factor for High Mg^2+^ Tolerance of *Staphylococcus aureus*

**DOI:** 10.3389/fmicb.2016.01888

**Published:** 2016-11-25

**Authors:** Joshua Armitano, Peter Redder, Vanessa A. Guimarães, Patrick Linder

**Affiliations:** Department of Microbiology and Molecular Medicine, CMU Faculty of Medicine, University of GenevaGeneva, Switzerland

**Keywords:** magnesium tolerance, *Staphylococcus aureus*, MpfA, CNNM, CorB, CorC, CBS

## Abstract

Internal bacterial concentration of Mg^2+^, the most abundant divalent cation in living cells, is estimated to be in the single millimolar range. However, many bacteria will thrive in media with only micromolars of Mg^2+^, by using a range of intensely studied and highly efficient import mechanisms, as well as in media with very high magnesium concentration, presumably mediated by currently unknown export mechanisms. *Staphylococcus aureus* has a particularly high Mg^2+^ tolerance for a pathogen, growing unimpaired in up to 770 mM Mg^2+^, and we here identify SA0657, a key factor in this tolerance. The predicted domain structure of SA0657 is shared with a large number of proteins in bacteria, archaea and even eukarya, for example CorB from *Salmonella* and the human CNNM protein family. One of the shared domains, a CBS pair potentially involved in Mg^2+^ sensing, contains the conserved Glycine326 which we establish to be a key residue for SA0657 function. In light of our findings, we propose the name MpfA, Magnesium Protection Factor A, for SA0657.

## Introduction

Mg^2+^ is the most abundant divalent cation present in living cells (Maguire and Cowan, 2002). It is critical in virtually all biological processes ranging from DNA replication and transcription to membrane stability and energy metabolism. Mg^2+^ cannot diffuse freely through membranes and therefore has to be imported through dedicated channels, using the electrochemical gradient of this ion (Payandeh et al., 2013). On the molecular level this transport is best understood in prokaryotes, where import of Mg^2+^ has been linked to four classes of transporters: CorA, MgtA/B, MgtE, and a recently described Nramp-related transporter (Kehres and Maguire, 2002; Groisman et al., 2013; Shin et al., 2014). Most bacteria possess several Mg^2+^ transporters and sometimes several from one class. CorA and MgtE are the most widespread transporters and considered the primary transporters of Mg^2+^ in bacteria (Papp-Wallace and Maguire, 2008). These Mg^2+^ channels can sense the internal Mg^2+^ concentration and close accordingly to regulate the influx (Payandeh et al., 2013). However, this regulation is apparently not sufficient to maintain internal Mg^2+^ concentration within acceptable ranges and bacteria therefore also carry systems for exporting Mg^2+^ (Gibson et al., 1991). All previously described prokaryotic transporters are involved in Mg^2+^ import, although when Gibson et al. (1991) looked for additional members of the *cor* (cobalt resistance) system of *Salmonella enterica* Typhimurium they noted that although CorA alone is necessary and sufficient for influx of Mg^2+^, eﬄux requires the presence of a co-effector, either CorB, CorC or CorD (Gibson et al., 1991). However, in the 25 years since this publication, no additional light has been shed on the functions of these proteins. Therefore, while Mg^2+^ import is relatively well understood, knowledge on Mg^2+^ export remains cryptic in prokaryotes.

The mechanisms of import and export presumably work together to maintain an optimal internal Mg^2+^ concentration. However, a recent study underlined that the tolerance for external Mg^2+^ varies considerably between bacterial pathogens, with for example *S.* Typhimurium being growth inhibited at only 285 mM MgCl_2_, whereas growth of *Staphylococcus aureus* remained uninhibited up to 770 mM MgCl_2_ (Cebrián et al., 2014). Genome annotation shows that *Staphylococcus aureus* possesses an *mgtE*-like (SA0867), two *corA*-like genes (SA2137 and SA2166), and two homologs of *Salmonella corB* (SA0657 and SA0780), none of which have been studied.

*Staphylococcus aureus* is a gram-positive opportunistic pathogen that is present in the nasal cavities of approximately 1/3 of the population (Kuehnert et al., 2006). It is one of the most frequent causes of nosocomial infections (Lowy, 1998; Wertheim et al., 2004) and can cause persistent infections due to its capability to develop biofilms (Götz, 2002; Bhattacharya et al., 2015) and Small Colony Variants (Proctor et al., 2006). The adaptation to these very different lifestyles requires fine-tuned regulation systems at every level from gene to protein. Our lab focuses on the study of the RNA helicases of the DEAD-box family which are important regulators of RNA metabolism and involved in ribosome biogenesis, RNA decay and translation regulation (Redder et al., 2015). *S. aureus* possesses two DEAD-box helicases, CshA and CshB and very little is known about the latter. We previously showed that a *ΔcshB* mutant strain is cold-sensitive (Redder and Linder, 2012), and here we also identify a growth defect on serum and a synthetic medium.

In the present study, we investigate the role of the *S. aureus* StCorB ortholog, SA0657, which we identified in a screen for suppressor mutations of the slow growth on synthetic medium of a *ΔcshB* mutant strain. We show that SA0657 is involved in Co^2+^ and Mn^2+^ sensitivity, and is a key element in detoxification of Mg^2+^, which prompts us to propose it as a Mg^2+^ exporter.

## Materials and Methods

### Strains, Media and Growth Conditions

Strains and plasmids used in this study are described in Supplementary Table S1. Construction of mutants was performed by allelic replacement as previously described, using the pyrEF/5-FOA counter selection system (Redder and Linder, 2012). *Escherichia coli* DH5α was grown in LB medium, supplemented when necessary with 100 mg/l ampicillin (Sigma-Aldrich, Buchs, Switzerland). *Staphylococcus aureus* was grown in Mueller-Hinton (MH) broth (211443, BD Biosciences, Allschwil, Switzerland) always supplemented with 10 mg/l uracil due to purine auxotrophy (Redder and Linder, 2012). Alternatively, *S. aureus* was grown in RPMI-1640 medium buffered with HEPES (Sigma R7388) and supplemented with 10 mg/l uracil. When necessary, medium was supplemented with 10 mg/l chloramphenicol (MHC), 10 mg/l erythromycin (MHE), 2 mg/l tetracycline (MHT), or 200 mg/l 5-fluoroorotate (MHFOA; US Biological, Swampscott, MA, USA). For serum experiments, *S. aureus* was grown in fetal calf serum (P3015-05, Pan-biotech, Aidenbach, Germany) supplemented with 10 mg/l uracil. For plates, agar was added at a final concentration of 13 g/l.

### Suppressor Mutant Selection

50 μl of overnight cultures of *ΔcshA/ΔcshB* or *ΔcshB* in RPMI medium, seeded from independent colonies, were plated on RPMI plates and grown at 37°C for 40 to 48 h. The biggest colonies were restreaked and isolated on RPMI medium twice and then grown in MH. To ensure all mutants were truly independent, only a single clone per initial culture was conserved (**Table 1**).

**Table 1:** The ten spontaneous *ΔcshB* suppressors with mutations in *SA0657*.

Series	#	Sequencing^a^	Mutation type	Allele^b^	Exact mutation on chromosome^c^
1	1	WGS	Frameshift	Y141^∗^	4 nt deletion at position 752104-7
	2	WGS	Point mutant	E183^∗^	G to T at position 752229
	3	WGS	Frameshift	I32IfsX7	1 nt deletion at position 751777
2	4	SS	Frameshift	I199WfsX25	7 nt insertion (GTGGAGA) at position 752275
	5	SS	Point mutant	G326C	G to T at position 752658
	6	SS	Frameshift	V21CfsX12	1 nt insertion (C) after position 751375
	7	SS	Frameshift	Q248NfsX26	Loss of 1 nt (C) at position 752424
	8	SS	Point mutant	G326D	G to A at position 752659
	9	SS	Double point mutant	P292S M330R	C to T at position 752555 and T to G at position 752671
	10	SS	Frameshift	R33EfsX8	Loss of 1 nt (T) at position 751778

*^a^WSG indicates Whole Genome Sequencing (by Illumina technology) and SS designates that the *SA0657* locus was amplified by PCR, and that the product was Sanger Sequenced. ^b^The allele description of frameshift mutant is as suggested (den Dunnen and Antonarakis, 2000). Shortly, the first mutated amino acid is indicated, followed by “fs” to indicate a frameshift and Xnn indicates the number of residues added before a stop codon. For example, R33EfsX8 indicates the arginine in the 33rd position is replaced by a glutamate followed by eight amino acids before a stop codon. ^c^The indicated genomic positions are according to the *S. aureus* N315 genome sequence. ^∗^ represents stop codons.*

### q-RT-PCR

For RNA extraction, bacterial cultures were grown to OD_600_ = 0.4 and harvested in ethanol:acetone (1:1). Samples were then washed in TE buffer (10 mM TRIS-HCl, 1 mM EDTA [pH 8.0]) and lysed in TE containing lysostaphin (250 μg/ml; Ambi products, Lawrence, NY, USA) for 10 min at 37°C. Total RNA was then extracted using the ReliaPrep RNA tissue miniprep system (Promega, Dübendorf, Switzerland) according to the manufacturer’s instructions. An additional DNase treatment (RQ1, Promega) was performed and RNA was subsequently extracted by phenol/chloroform extraction and finally concentrated by ethanol precipitation. mRNA levels were quantified by quantitative RT-PCR using GoTaq 1-Step RT-qPCR kit (Promega). Primers were designed using Primer3Plus^1^ and obtained from Microsynth (Balgach, Switzerland; Supplementary Table S1). Reactions were performed using 4 ng of RNA in 10 μl with 0.2 μM of primers (Supplementary Table S1). Reactions were performed as follows: 15 min at 37°C followed by 10 min at 95°C and then 40 cycles of 10 s 95°C, 30 s 60°C and 30 s 72°C. Levels of mRNA of target genes are estimated relative to the level of hu (SA1305) for each sample (Oun et al., 2013). Measurements were performed in triplicates and on two independent cultures.

### Sequencing and Bioinformatics

Whole genome sequencing (WGS) was performed at the iGE3 Unige Genomics platform. Sanger sequencing was performed by Fasteris (Fasteris SA, Switzerland). Multiple alignments were performed using Clustal Ω^2^, and boxshade^3^. Ortholog analysis relied on the OMA database^4^ (Altenhoff et al., 2015).

## Results

### Slow Growth of a *ΔcshB* Mutant on Serum and Artificial Serum-Substitute Medium Can be Suppressed by Mutations in SA0657

**FIGURE 1:**
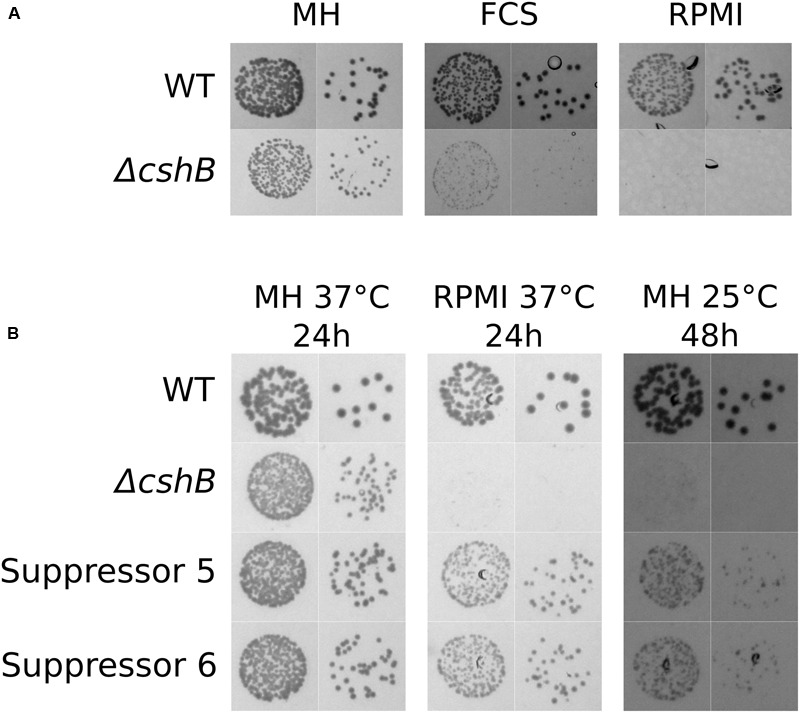
***ΔcshB* grows poorly on serum and RPMI.** Two dilutions of overnight cultures (10^-5^ and 10^-6^) of each strain were spotted on either MH-, RPMI- or FCS (Fetal Calf Serum)-agar, and incubated at the indicated temperatures and times. **(A)** A *ΔcshB* strain grows poorly on FCS and its synthetic substitute RPMI after 20 h of incubation at 37°C. **(B)** Two examples of spontaneous suppressor mutants show improved growth both on RPMI and on MH at 25°C. The strain numbers are indicated, see **Table 1** for details.

The pathogenicity of an opportunistic pathogen like *Staphylococcus aureus* is clearly dependent on its ability to grow at conditions that are encountered during infection. The ability to use the metabolites found in serum to support growth is probably crucial for sepsis, one of the most serious Staphylococcal infections. We have found that, in addition to the previously described cold-sensitivity (Redder and Linder, 2012), a deletion mutant of the DEAD-box helicase *cshB* grows poorly on Fetal Calf Serum (**Figure 1A**). We observed a similar growth defect on RPMI, a defined medium containing all 20 amino-acids, vitamins, salts and glucose, which is used as a serum substitute to allow *in vitro*-growth of mammalian cells (**Figure 1A**). The growth deficiency in RPMI suggests that the effect observed in serum is possibly due to one or several missing components, rather than an inhibitory activity present in the serum. To better understand what causes the *ΔcshB* mutant to grow poorly on RPMI medium (and serum), we isolated spontaneous suppressor mutants. Briefly, we plated independently grown *ΔcshBΔcshA* cultures on RPMI-agar plates and selected colonies that appeared first and were bigger than the average colonies on the same plate. A *ΔcshBΔcshA* strain was chosen as parental strain to exclusively detect mutations that would “directly” suppress the loss of CshB, and avoid mutations that could allow CshA to take over the functions of CshB. This approach was possible because growth of a *ΔcshA* strain is not inhibited on RPMI-agar (Supplementary Figure S1). To identify the exact suppressor mutations, WGS was performed on three of the spontaneous mutants. Remarkably all three suppressor mutants had mutations that resulted in stop-codons inside the SA0657 open reading frame (ORF; **Table 1**), encoding a hypothetical protein with homology to the *Salmonella* gene St*corB* (see below). Intrigued by the fact that all mutations occurred in the same gene, we isolated a further 12 independent *ΔcshB* suppressor mutants (Suppressor series 2), and amplified the SA0657 locus by PCR and Sanger-sequenced the products. This analysis revealed that 7 out of these 12 additional suppressor mutants harbored mutations in SA0657. Thus, out of the 15 analyzed suppressor strains, 10 were mutated in SA0657 (**Table 1**). The growth phenotypes of these 10 spontaneous suppressor strains were assessed at 37°C and 25°C on MH medium as well as on RPMI medium at 37°C (examples shown in **Figure 1B**), and as expected, all selected suppressor mutants exhibit improved growth on RPMI medium compared to *ΔcshB.* Moreover, these mutations intriguingly also slightly improve growth at 25°C in MH medium (**Figure 1B**), indicating that they act high up in the hierarchy of *ΔcshB* defects.

### mRNA Expression of SA0657 Depends Both on Growth Conditions and on CshB

**FIGURE 2:**
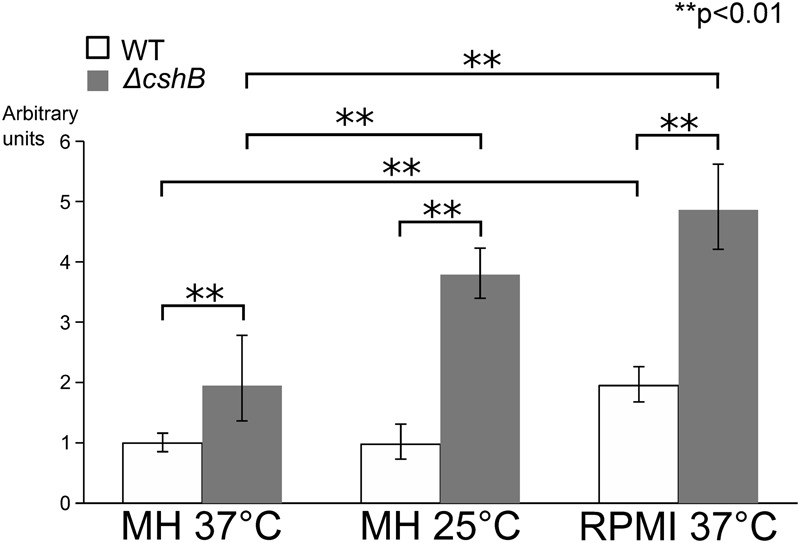
**SA0657 mRNA is overexpressed in a *ΔcshB* background.** Total RNAs were extracted from exponentially growing cells in the following conditions: MH medium at 37°C, MH medium 25°C and in RPMI medium at 37°C, each in biological duplicates. mRNA levels were determined by qRT-PCR and normalized to a stably expressed control gene (hu: SA1305). Expression is shown in arbitrary units, where the WT level at 37°C is set to 1. Unpaired *t*-test was performed to evaluate statistical significances. ^∗∗^ indicates a *p*-value lower than 0.01.

Since CshB is a predicted DEAD-box RNA helicase and therefore is likely to be involved in the regulation of RNAs, we tested whether *SA0657* mRNA levels were affected in a *ΔcshB* strain. The mRNA levels of *SA0657* were determined in the WT and *ΔcshB* strains, grown at 37°C in MH and RPMI medium and 25°C in MH medium. This was possible because the *ΔcshB* strain does grow in RPMI and at 25°C in MH, albeit with doubling times approaching 2 h, compared to the doubling times of about 45 min for the WT strain in those conditions. The results show that *SA0657* expression is significantly higher in the *ΔcshB* strain than in the WT in all tested growth conditions, and this overexpression is even more striking under growth-limiting conditions (RPMI and 25°C; **Figure 2**). In the WT, even though *SA0657* levels are slightly higher in RPMI than in MH at 37°C, *SA0657* levels are significantly lower in each condition compared to the *ΔcshB* strain.

### Only Loss-of-Function Mutations Are Isolated in SA0657

**FIGURE 3:**
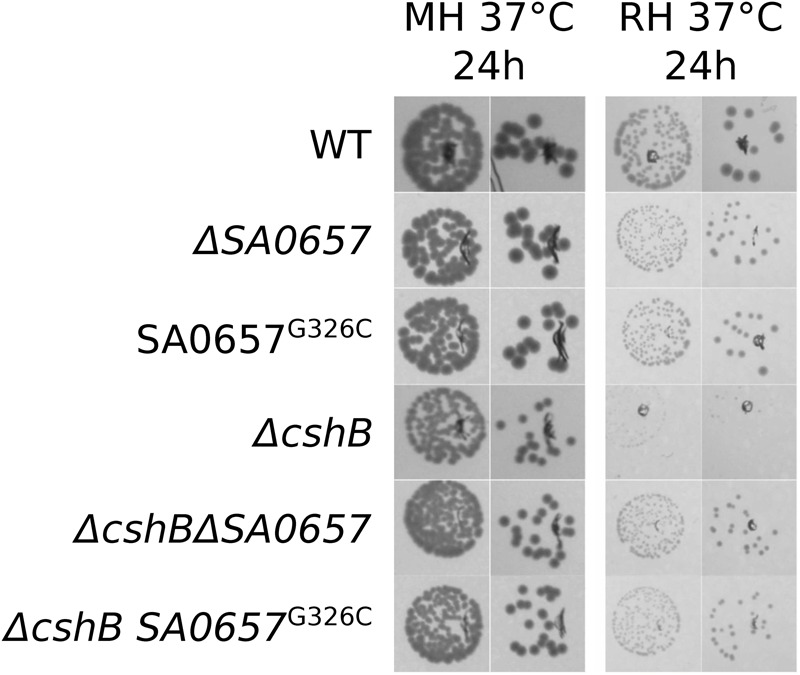
**Reconstruction of *ΔSA0657* and *SA0657*^G326C^ mutations confirm the suppression of *ΔcshB* slow growth on RPMI.** Two dilutions from overnight cultures (10^-5^ and 10^-6^) of each strain were spotted on MH- or RPMI-agar, and incubated at 37°C for 24 h. A *ΔcshB* strain grows poorly on RPMI, while growth can be restored by deletion of *SA0657* or the *SA0657*^G326C^ allele.

Most of the suppressor mutations in SA0657 are frameshifts (V21CfsX12, I32IfsX7, R33EfsX8, I199WfsX25, Q248NfsX26) or stop codons (Y141*, E183*; **Table 1**). Some of the predicted truncated proteins are extremely short (V21CfsX12, I32IfsX7 and R33EfsX8), essentially abolishing the presence of SA0657. The longest truncated version of SA0657 maintains about half of the protein intact, corresponding to the predicted transmembrane domain (see below) and about 80 additional amino acids (AA), revealing that the expression of the transmembrane domain by itself, if correctly expressed, induces no phenotype in a *ΔcshB* background. Interestingly, we also identified two different point mutations leading to the change of the same Gly326 residue (G326C and G326D). Based on the nature of the mutations that result in drastically truncated proteins, we believe that all ten identified mutations inactivate the function of SA0657. To confirm that the detected mutations were indeed responsible for the suppression, we constructed a deletion mutant of *SA0657* (strain *ΔSA0657*, where only the first 25 codons remain) and recreated the *SA0657*^G326C^ allele in both wild-type and *ΔcshB* backgrounds. As expected, the deletion of *SA0657* and the mutated allele of *SA0657* are both able to suppress the slow growth of *ΔcshB* as it is the case of the spontaneous mutants (**Figures 1, 3** and Supplementary Figure S2).

**FIGURE 4:**
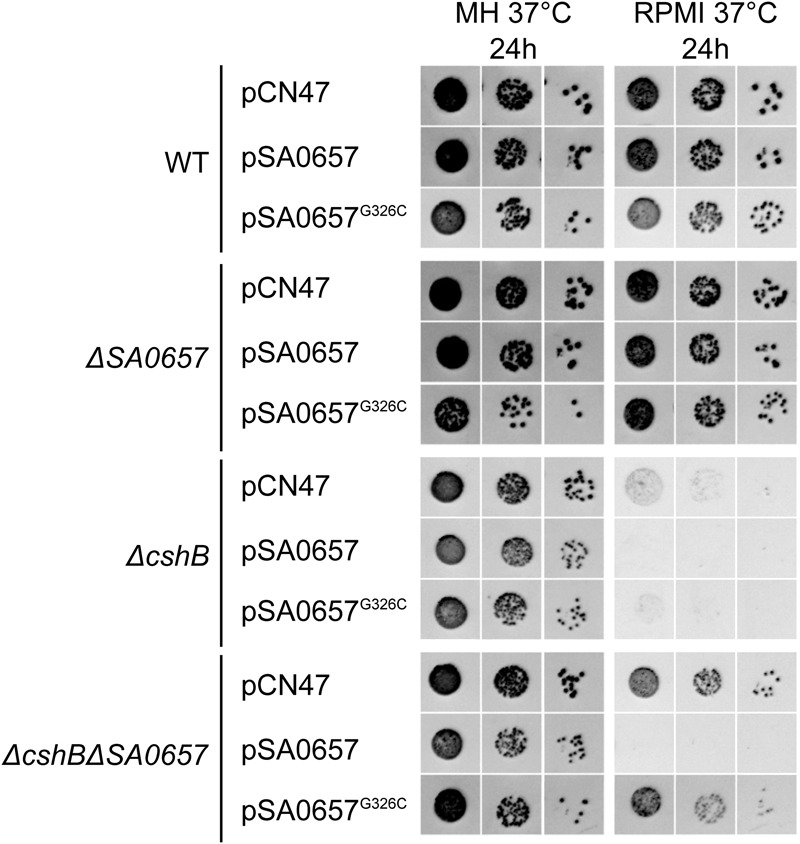
**Complementation of *ΔSA0657* abrogates the *ΔcshB*-suppressing effect.** Three dilutions (10^-4^, 10^-5^ and 10^-6^) of overnight cultures of each strain were spotted on MH- or RPMI-agar, supplemented with 10 μg/ml erythromycin, and incubated at 37°C for 24 h. The different strains carry either an empty plasmid (pCN47), a plasmid carrying a WT allele of *SA0657* under control of its own promoter (pSA0657) or a mutated allele *SA0657*^G326C^ (pSA0657^G326C^). The *ΔcshB* pCN47 strain grows poorly on RPMI and growth is even slower in presence of pSA0657. The *ΔcshBΔSA0657* pCN47 grows on RPMI while growth in inhibited in presence of pSA0657 but not in presence of pSA0657^G326C^.

We also performed complementation experiments on the reconstructed strains. We constructed WT and G326C alleles of *SA0657* with C-terminal flag-tags, and expressed them on a low copy number vector [pCN47 ∼20 to 25 copies/cell (Charpentier et al., 2004)] under control of the native *SA0657* promoter. As expected, the presence of the WT construct, pSA0657, abolished the suppressor phenotype when transformed into *ΔcshB*/*ΔSA0657*, whereas this was not the case for the mutant construct and the empty vector (pSA0657^G326C^ and pCN47, respectively; **Figure 4**). The flag-tag on WT and mutant SA0657 was then used in a western blot, and revealed similar expression levels for the two alleles, which excludes that the G326C mutation merely led to poor expression (or rapid proteolysis) of SA0657^G326C^ (Supplementary Figure S3). This confirmed that the SA0657^G326C^ mutation is solely responsible for the suppressor effect, and not dependent on a hypothetical undetected secondary mutation in the genomes of the suppressor strains.

Although we have not sequenced the entire genomes of all suppressor strains, the reconstruction of the deletion and the point mutation and their complementation, suggest that the identified mutations in SA0657 are responsible for the suppressor phenotype in each case. The *ΔSA0657* and SA0657^G326C^ mutations by themselves in a *cshB+* background do not cause any growth phenotype in the tested conditions, MH 37°C, 25°C and RPMI (**Figures 1** and **3**).

### Mutations in SA0657 Do Not Suppress Δ*cshA*

The presence or absence of CshA has no influence on SA0657 mutations ability to restore growth of *ΔcshB* on RPMI as shown by the appearance of *SA0657* spontaneous mutations in both *ΔcshB* and *ΔcshAΔcshB* strains. While *ΔcshA* grows as WT on RPMI, the cold-sensitivity phenotype of *ΔcshB* is shared by *ΔcshA*, and we therefore tested whether *ΔSA0657* could suppress the latter. A *ΔcshAΔSA0657* strain was constructed, but it did not grow better than *ΔcshA* at 25°C (Supplementary Figure S1). The triple mutant *ΔcshA*/*ΔcshB*/*ΔSA0657* did not grow either at 25°C, but as expected, did grow on RPMI-agar due to the suppression of *ΔcshB* (Supplementary Figure S1).

### SA0657 has a Predicted Transmembrane Region and a Crucial CBS Pair in a Universally Conserved Layout

**FIGURE 5:**
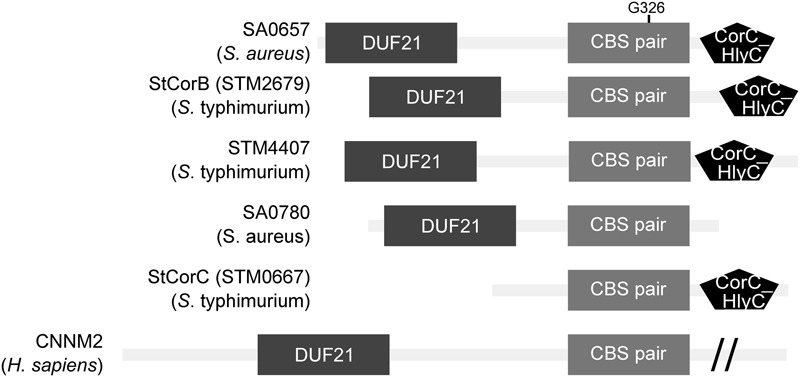
**The extended family of SA0657-like proteins exhibit a universally conserved architecture.** The predicted domains of SA0657 and diverse related proteins are shown schematically. The Gly326 is located in the CBS pair, a domain present in all these homologs. Except for StCorC, all proteins possess a DUF21 (membrane anchor) domain.

SA0657 encodes a protein of 449 AA, predicted to be composed of a transmembrane region in the N-terminal part (DUF21: IPR002550), followed by a pair of CBS domains and a CorC_HlyC domain of unknown function in the C-terminal part (**Figure 5**). CBS domains (IPR000644) have poor sequence similarity but are defined by a conserved structure and are found in pairs that are referred to as a “Bateman module.” The binding of adenosine nucleotides (AMP, ATP, S-adenosylmethionine) or Mg^2+^ triggers conformational changes of the protein leading to change in activity (Baykov et al., 2011; Ereño-Orbea et al., 2013), and a CBS pair is, for example, responsible for the regulation of the activity of the magnesium transporter MgtE (Imai et al., 2012).

The OMA orthology database describes 2043 proteins as orthologs of SA0657, among which is *Salmonella* CorB (StCorB: STM2679, see Supplementary Figure S5) (Altenhoff et al., 2015)^5^. These two proteins share the same domains architecture but their overall similarity is low (22%; **Figure 5** and Supplementary Figure S7). StCorB was identified in a screen for *S*. Typhimurium mutants resistant to cobalt (Gibson et al., 1991), where the authors proposed CorB to be an additional factor necessary for Mg^2+^ eﬄux (along with two other proteins CorC and CorD), while influx was suggested to be solely mediated by CorA. Among the SA0657 orthologs, we identified a paralog of CorB in *S.* Typhimurium genome (STM4407), whose function is unknown and which was not identified in the cobalt resistance screen by Gibson et al. (1991). The *S. aureus* genome additionally encodes SA0780, a paralog to SA0657 that is 103 residues shorter (**Figure 5** and Supplementary Figure S7). To get a better grasp of the evolution and the distribution of SA0657 homologs in the genomes of the OMA database, we performed a phylogenetic analysis of the 3743 predicted orthologs of SA0657, SA0780, StCorB, StCorC, STM4407, human CNNM2 and murin CNNM2 (see Discussion) (Supplementary Figure S4). This analysis reveals that SA0657 orthologs are widely distributed in the prokaryotic kingdom and that each phylum often possess several paralogs of SA0657, most often three, with up to nine in the *Streptomyces coelicolor* genome (Supplementary Figure S6). This suggests that the SA0657 ancestor arose long before phyla separation. Proteobacterial proteins, StCorB (STM2479), StCorC (STM4407) and STM4407 are present in different clusters each counting several hundreds leaves, and SA0657 belong to a cluster of 273 Firmicute proteins. Its paralog, SA0780, differs significantly and is found, according to our tree, only in Staphylococcal genomes.

The G326 residue of SA0657, which we identified as essential for the function of SA0657 (**Figure 4**), is located in the second CBS domain of the predicted CBS pair (**Figure 5**). This glycine residue that corresponds to the 40th position of the cannon CBS domain and belongs to the ribose phosphate-binding motif, is extremely conserved according to the CBS logo plot unlike the other residues (PS51371^6^). This marks the importance of this glycine, and the substitution of this particular amino-acid most likely inactivates the CBS domain and consequently the activity of SA0657. Interestingly, in SA0780 the equivalent of Gly326 from SA0657 is changed to an alanine. This is not a recent mutation, since it is also found in *Staphylococcus epidermidis* and *Staphylococcus carnosus*, suggesting that SA0780 may have evolved to fulfill a different function than SA0657.

### SA0657 Protects the Cell from High Extracellular Magnesium Concentrations: Mutants are Inhibited by as Little as ∼10 mM Mg^2+^

**FIGURE 6:**
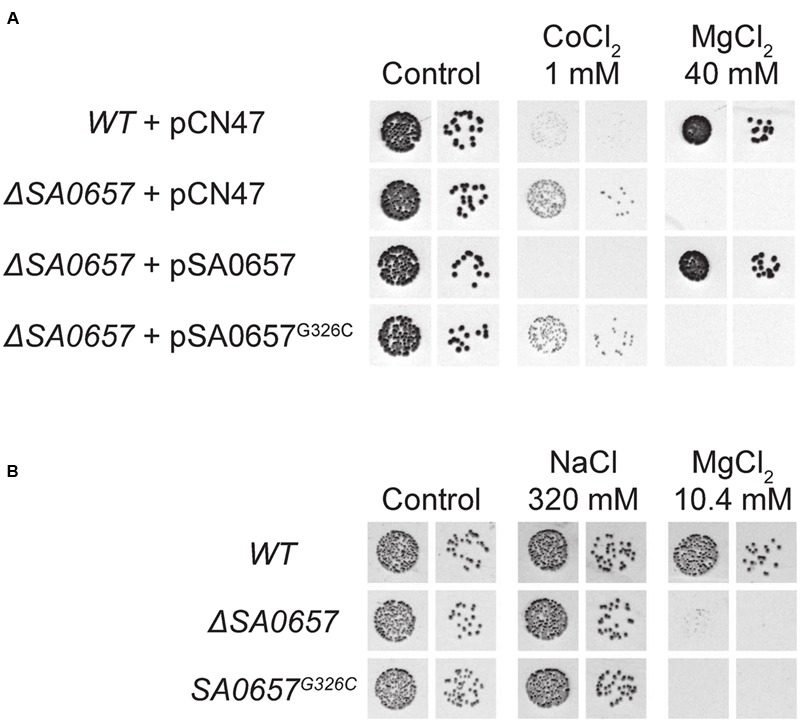
**Co^2+^ resistance and Mg^2+^ sensitivity of SA0657 mutants. (A)** MH-agar plates were complemented with 10 mg/l erythromycin and the indicated amount of salt (the control plate had no added salt). Two dilutions (10^-5^ and 10^-6^) of overnight cultures of each strain were spotted. The drops deposited on the surface of MgCl_2_ plates do not flow out, making the spots appear smaller even though the same volume of culture was spotted each time. Also note that since *S. aureus* is naturally much more resistant to Co^2+^, we used much higher Co^2+^ concentrations than those used by Gibson et al. (1991). **(B)** RPMI medium was complemented with salt to reach the final concentration indicated for the cation (feasible, because of the defined composition of RPMI medium).

Since SA0657 is an ortholog of StCorB, originally described in a study of *Salmonella* mutants with increased tolerance for Co^2+^ ions (Gibson et al., 1991), we also tested Co^2+^ resistance of the *ΔSA0657* mutant (albeit with the much higher Co^2+^ concentrations that are appropriate for *S. aureus*). This revealed a clear growth difference between WT and *ΔSA0657*, when 1 mM CoCl_2_ was added to the MH plates, which was fully complemented by a plasmid expressing SA0657, but not by a plasmid with SA0657^G326C^ (**Figure 6A**). Interestingly, the resistance was also observed for MnCl_2_ (Supplementary Figure S8). Since domain predictions and ortholog analyses suggested that SA0657 can recognize Mg^2+^ and has a membrane spanning domain, we suspected that SA0657 was involved in removing excess Mg^2+^ ions from the cell. To test this hypothesis, we also examined the growth of the *ΔSA0657* and WT strains at elevated Mg^2+^ concentrations. Whereas the WT with an empty erythromycin-resistance plasmid grew normally on rich medium with 40 mM MgCl_2_, growth of the *ΔSA0657* strain with the same empty plasmid was completely abrogated, unless a SA0657-expressing plasmid was used for complementation (**Figure 6A**). The growth-inhibition was specifically due to the Mg^2+^ ions, since addition of 320 mM NaCl was permissive for growth of both SA0657 mutant and WT strains (**Figure 6B**), showing that neither the ionic strength of the medium, nor the concentration of Cl^-^ ions are inhibitory. Additionally, we obtained similar results whether we used MgCl_2_ or MgSO_4_ (Data not shown). Deletion of SA0657 in a different *S. aureus* strain, RN4220, leads to the same Mg^2+^ sensitivity, highlighting that the role of SA0657 is not strain specific (Supplementary Figure S9A). A true inhibitory concentration is difficult to determine using a complex medium such as MH, so we decided to use the defined RPMI medium (which normally contains 0.4 mM Mg^2+^) as basis for defining the lower end of the inhibitory concentrations. Intriguingly a final Mg^2+^ concentration of only 10.4 mM was sufficient to inhibit growth on RPMI plates (**Figure 6B**), as opposed to ∼40–80 mM on MH-plates.

We furthermore tested whether this phenotype extended to the SA0780 deletion mutant, however, no Mg^2+^ sensitivity was observed, even with 80 mM MgCl_2_ added (Supplementary Figure S9B) further underlining the functional differences between SA0657 and SA0780. The *Δ0780* mutation by itself slows the bacterial growth significantly, and therefore *SA0780* cannot be a non-functional pseudogene (Supplementary Figure S9B). A double *ΔSA0657Δ0780* deletion mutant thus combines the slow growth with sensitivity to 80 mM MgCl_2_, and we detected no additional effects in this double mutant.

## Discussion

*Staphylococcus aureus* is highly resistant to divalent cations, with a maximal non-inhibitory Mg^2+^ concentration of 770 mM, ∼4.5 times higher than for *Escherichia coli* and twice that of *Listeria monocytogenes* (Cebrián et al., 2014). We have shown that SA0657 plays an essential role in this resistance, since as little as 10.4 mM Mg^2+^ inhibits growth of *SA0657* deletion mutants in a defined medium (**Figure 6B**), a phenotype that could also be linked to point mutations at the crucial Gly326 CBS domain residue identified in this study. We have additionally observed that when *SA0657* is deleted, *S. aureus* is mildly resistant to Co^2+^ and Mn^2+^ ions, albeit at concentrations that presumably are rare or non-existent in its natural environment (**Figure 6** and Supplementary Figure S8). Taken together, it seems likely that the main function of SA0657 is in the regulation of intra-cellular Mg^2+^ levels, and we therefore propose to rename the gene *mpfA* (for magnesium protection factor A).

In addition to the increased resistance to cobalt, MpfA has all the characteristics of the originally identified *Salmonella* StCorB, such as the predicted membrane domain in the C-terminal part (DUF21), the CBS pair with the conserved glycine and a C-terminal CorC_HlyC domain. A phylogenetic analysis of MpfA and StCorB orthologs showed that these proteins are present in many organisms and that often several copies are present. Nevertheless in some cases, as for the *S. aureus* CorB/MpfA paralog (SA0780) the proteins may have different functions. Indeed, our results show that SA0780 does not reveal the same phenotypes as SA0657 if deleted. Moreover, the highly conserved G326 residue is replaced by an alanine in SA0780 (Supplementary Figure S7). Since the *ΔSA0780* mutant grows significantly slower than the WT strain, and *SA0780* is conserved in other *Staphylococcus* species, we believe that it is not a pseudogene but that it has another, yet to be identified, function.

### MpfA is Related to an Eukaryotic Family of Mg^2+^ Transporters

At this stage it is difficult to draw a firm conclusion on the molecular function of MpfA. Nevertheless, the similarity with eukaryotic Mg^2+^ transporters is intriguing. In eukaryotes, eight different types of magnesium transporters have been described, and several of these are distant relatives of bacterial transporters (Quamme, 2010). Among those, a family of proteins called CNNM (cyclin M family) shows similarity with bacterial CorC/CorB (**Figure 5**) (Wang et al., 2004). The CNNM proteins are membrane anchored by a DUF21 domain in their N-terminal part and regulate their activity depending on Mg^2+^ levels perceived by their CBS pair (Wang et al., 2004; Corral-Rodríguez et al., 2014). A rare genetic disease (MIM:607803) conferring hypomagnesaemia, i.e., low Mg^2+^ level in the blood leading to tetany, seizure and cardiac arrhythmia, has been mapped to mutations in CNNM2, which is involved in Mg^2+^ excretion in kidney (Goytain and Quamme, 2005; Hirata et al., 2014). One of these mutations is a single amino acid substitution in the CBS pair that renders CNNM2 constitutively active regardless of the Mg^2+^ concentration, leading to continuous excretion of Mg^2+^ into the urine (Stuiver et al., 2011; Corral-Rodríguez et al., 2014). This point mutation is located only three amino acid residues from the glycine residue which is equivalent to Gly^326^ of MpfA (**Figure 5**), which highlights the fundamental importance of this part of the CBS pair in regulating the activity of these proteins, since the mutation of the threonine renders CNNM2 constitutively active, whereas that of the glycine renders MpfA inactive.

### Putative MpfA-Mediated Mg^2+^ Protection Mechanism

Our *mpfA* mutants are hypersensitive to Mg^2+^, indicating that the homeostasis of this otherwise well tolerated cation, is perturbed. Three potential (and not mutually exclusive) mechanisms can be imagined for keeping the intracellular free Mg^2+^ concentration down. One possibility would be to sequester the unwanted Mg^2+^ to remove it from the pool of free Mg^2+^. However, this mechanism is highly impractical for abundant ions such as Mg^2+^, since the concentration of binding-protein needed would be very high. A second possibility is to efficiently keep excess Mg^2+^ outside the cell, and avoid importing more than is required. The main Mg^2+^ import proteins, CorA and MgtE, do have built-in sensors that constrict the transport channel and prevent further import when internal Mg^2+^ levels are too high (Moomaw and Maguire, 2008). However, at least in *Salmonella*, this closing mechanism is not completely leak-proof, since a *corA mgtA mgtB* triple mutant, theoretically incapable of importing Mg^2+^, is viable in growth medium with high Mg^2+^ concentration, suggesting that the bacteria can obtain the essential Mg^2+^ ions via channels that are meant for other purposes (Hmiel et al., 1989). The third possibility is that excess Mg^2+^ is exported from the cell, a process that would need to go against the electrochemical potential and therefore require energy. Virtually nothing is known about bacterial Mg^2+^ export, and since no radioactive isotopes are currently commercially available, it is unfortunately very difficult to address this question.

Nevertheless, in analogy to the similarity (albeit weak) with eukaryotic CNNM proteins, the N-terminal transmembrane domains, and the Mg^2+^ sensitivity of the *ΔmpfA* and *mpfA*^G326^ mutants, we propose that MpfA is either a Mg^2+^ exporter itself or promotes Mg^2+^ export via another protein. If our hypothesis is correct then the Mg^2+^ concentration within the cell will increase in absence of MpfA, and will exert the mild Co^2+^ and Mn^2+^ resistance exhibited by improved competition against these divalent cations for binding sites. Furthermore, it is also possible that the higher Mg^2+^ concentration more efficiently closes Co^2+^ and Mn^2+^ import routes.

### What is the Role CshB?

Inactivation of MpfA can partially suppress the strikingly slow growth of a *ΔcshB* mutant on Fetal Calf Serum, RPMI, or at 25°C (**Figure 1**). qRT-PCR experiments showed that in absence of CshB the mRNA steady state levels of *mpfA* in RPMI and at 25°C are significantly increased (although possibly via an indirect effect; **Figure 2**). CshB is a DEAD-box protein, a family of enzymes that are mainly known for unwinding short RNA duplexes but that can in some instances also be involved in annealing or strand exchange (Chamot et al., 2005). At present we cannot distinguish between the hypotheses where the overexpression of MpfA in absence of CshB leads to cold sensitivity or where a change in intracellular magnesium concentration changes the stability of RNA-RNA duplexes that would need CshB for annealing or unwinding.

### Model

**FIGURE 7:**
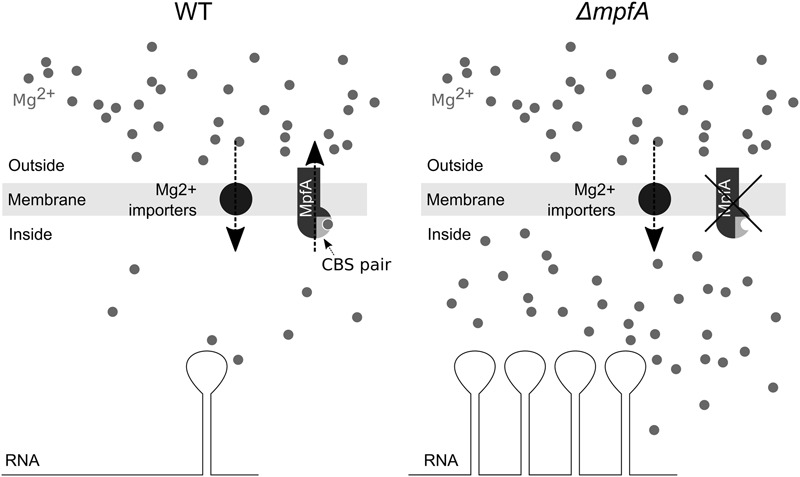
**Proposed model of MpfA function in high Mg^2+^ medium.** Mg^2+^ ions are represented as dots and the amount of dots corresponds to the concentration. The arrows represent the direction of Mg^2+^ transport, and we propose that MpfA (SA0657) is a Mg^2+^ exporter (or at least an essential component of one). In a WT strain **(Left)** internal Mg^2+^ concentration is maintained lower than the external one by the combined action of Mg^2+^ importers (MgtE/CorA) and MpfA. MpfA activity is potentially regulated by Mg^2+^ internal concentration, through binding of Mg^2+^ at the CBS pair. In absence of MpfA **(Right)**, Mg^2+^ cannot be exported anymore leading to an increase in internal Mg^2+^ up to toxic concentration. This toxicity could for example manifest itself in modified RNA structures, where higher Mg^2+^ leads to more stable RNA double helices.

The data presented here allow us to propose the following speculative model (**Figure 7**): *S. aureus* maintains internal Mg^2+^ levels within acceptable ranges, through the combined action of its importers, CorA and MgtE, and a proposed exporter MpfA. When grown in high Mg^2+^, MpfA activity is essential to maintain internal Mg^2+^ low enough for cells to grow. While the molecular causes rendering certain cations toxic, such as Co^2+^ or Ni^2+^, have been investigated (Beyersmann and Hartwig, 1992; Macomber and Hausinger, 2011), it is harder to explain how an ubiquitous and abundantly available ion such as Mg^2+^, can be toxic, especially considering the ∼10 mM inhibitory concentrations we observed (**Figure 6B**), which are not far from the estimated internal concentrations (Maguire and Cowan, 2002). However, Mg^2+^ importers are extremely efficient at importing Mg^2+^ even against its concentration gradient, which can lead to internal accumulation to concentrations significantly higher than that of the medium (Maguire, 2006). In case too much Mg^2+^ is imported, one could imagine that the accumulation of magnesium would become toxic through the increase in ionic strength; however, it seems unlikely that the contribution from Mg^2+^ can significantly impact the status quo generated by mono-valent cations Na^+^ and K^+^. Instead, the Mg^2+^ ions probably compete for the binding sites of other divalent cations. The toxicity of other divalent cations, such as Co^2+^ and Mn^2+^, is probably due to a similar mechanism, where they inactivate enzymes by substituting the native metals. This hypothesis is furthermore consistent with our observation that the *mpfA* mutant strains, which presumably have elevated internal Mg^2+^ concentrations, are more resistant to Co^2+^ and Mn^2+^ than the wild-type strain (**Figure 6** and Supplementary Figure S8). Mg^2+^ is involved in stabilizing secondary RNA structures (reviewed in Bowman et al., 2012), and a second potential mechanism of Mg^2+^ toxicity could be by making specific RNA structures excessively stable or inflexible. This could potentially block the activities of ribozymes and riboswitches and perhaps even pause the translational machinery. Although it is highly uncertain whether intra-cellular Mg^2+^ concentrations could change enough for a measurable effect on RNA structures, this explanation would fit with the suppression of *ΔcshB* we observe for the MpfA mutants. Indeed as a DEAD-box protein, CshB is probably involved in RNA structure resolving. A shift in the structures of the RNAs could very well relieve the need for the CshB enzyme, however, the explanation might be more complex, since the cold-sensitivity of the *ΔcshA* strain is not rescued by deletion of *mpfA* (**Figure 3**), and further investigation into the functions of CshA and CshB will be needed. Finally, it has previously been shown that membrane fluidity lowers with increasing cation, in particular Mg^2+^, concentration (Jajoo et al., 1994; Šegota et al., 2015).

## Conclusion

We identified SA0657 (*mpfA*) as an important actor in magnesium homeostasis in the opportunistic pathogen *S. aureus*. Based on the sensitivity to elevated magnesium concentration in the medium when MpfA is inactivated, we propose that it participates in export of the divalent cation.

## Author Contributions

JA, PR, and PL designed the experiments and wrote the manuscript. JA, PR, and VG performed the experiments.

## Conflict of Interest Statement

The authors declare that the research was conducted in the absence of any commercial or financial relationships that could be construed as a potential conflict of interest.

## Footnotes

1 http://www.bioinformatics.nl/cgi-bin/primer3plus/primer3plus.cgi

2 http://www.ebi.ac.uk/Tools/msa/clustalo/

3 http://mobyle.pasteur.fr/cgi-bin/portal.py#forms::boxshade

4 http://omabrowser.org/oma/home/

5 http://omabrowser.org/oma/home/

6 http://prosite.expasy.org/cgi-bin/prosite/sequence_logo.cgi?ac=PS51371
