# Supplementary material for: An Essential Factor for High Mg2+ Tolerance of Staphylococcus aureus
Source: Front Microbiol. 2016 Nov 25;7:1888. doi: 10.3389/fmicb.2016.01888 (PMC5122736; doi:10.3389/fmicb.2016.01888)
Supplement: Supplementary file 1 [file Table_1.pdf]

Bacterial strain or plasmid	Description	Short name	Source or reference
Bacterial strain			
E. coli DH5α	Standard cloning strain		Lab strain
S. aureus			
PR01	SA564RD Δ*pyrFE*	WT	Redder and Linder, 2012
PR01-09	PR01 ΔSA1387 (Parental strain for suppressors serie 2)	Δ*cshB*	Redder and Linder, 2012
PR01-15	PR01ΔSA1885::kan	Δ*cshA*	Linder et al., 2014
PR01-14	PR01-09 Δ1885::kan (Parental strain for suppressors serie 1)	Δ*cshA*Δ*cshB*	This work
PR01-30	PR01-09 ΔSA0657 ::catcat	Δ*cshB*Δ*mpfa*	This work
PR01-36	PR01 ΔSA0657::cat	Δ*mpfa*	This work
PR01-59A	PR01 ΔSA0780		This work
PR01-60A	PR01-09 ΔSA0780		This work
PR01 mpfa-G326C	PR01 SA0657-G326C		This work
PR01 ΔcshB mpfa-G326C	PR01Δ*cshB* SA0657-G326C		This work
PR01-41	PR01-15 ΔSA0657::cat	Δ*cshA*Δ*mpfa*	This work
PR01-31	PR01-14 ΔSA0657::catcat	Δ*cshA*Δ*cshB*Δ*mpfa*	This work
PR02	RN4220 Δ*pyrFE*		Redder and Linder, 2012
PR02Δmpfa	PR02 Δ*SA0657*		This work
Plasmid			
pCN47	Shuttle vector carrinyng pT181copwt repC and an erythromycin resistance cassette		Charpentier et al., 2004
pCN-SA0657-flag	pCN47 carrying SA0657 gene and its promoter (between coordinates 751430 and753032) fused to a flag tag in C-terminal, betwen restriction sites SalI and BamHI	pSA0657	This work
pCN-SA0657-G326C-flag	pCN-SA0657-flag with a single base subsitution (G to T in position 975 of the ORF)	pSA0657^{G326C}	This work

Primer name	Sequence
Hu-F	GGTTTCGGTAACTTTGAGG
Hu-R	CAGTTTGACGGTTACGACC
SA0657-qPCR-F2	ACGACGACGATTTCGTTTGC
SA0657-qPCR-R2	GCATCAGTTTGGGCATCAGG
SA0657-Sal-F1	TAATAAGTCGACAATAAAACAAGGCAAGGTTCAC
SA0657_flag_Bam_R	CGGGATCCTTACTTATCGTCGTCATCCTTGTAATCTTCTGATTTTTCATCTTCATCAGACTG
SA0657_G326C_DsFw	GTGGAACGGCATGTATTTTAACGATGGAAGATA
SA0657_G326C_UpRev	TATCTTCCATCGTTAAAATACATGCCGTTCCAC
SA0657-L-Nar-F1	CGAGAAGGCGCCATATTCATGGTGGTTATGGTC
SA0657-L-Bam-R1	AGGTTGGGATCCAACAAATACAGTGGTTAATGC
SA0657-R-Bam-F1	AGGTTGGGATCCGAGACCTACTATCGGACAGTC
SA0657-R-Xho-R1	CCACGGCTCGAGAAACAAGGAATGACGACTCT
SA0780-L-Bam-R1	TATTAAGGATCCAATGATCACGCGTGTAGTC
SA0780-Nar-F1	TAATAAGGCGCCAATGGGTATTGATACCATTTTG
SA0780-Xho-R1	TAATAACTCGAGGCTTTCCACTAAACGCTTTTG
SA0780-R-Bam-F1	TATTATGGATCCGAAGAATTATTAGGTATGGAAAT
ERY-Bam-F1	ACTATGGATCCTTTAAGAACTTTCTTTTTTTAC
ERY-Bgl-R1	TATCAAGATCTCACAAAAAATAGGCACACG
CAT-Bam-F1	GTATCAGGATCCATGTATTCTCAAGATAAGAAAG
CAT-Bgl-R1	GAGCATAGATCTTCTTCAACTAACGGGGC

Table S1: Strains, plasmids and primers used in this study

1. **Redder P, Linder P.** 2012. New Range of Vectors with a Stringent 5-Fluoroorotic Acid-ased Counterselection System for Generating Mutants by Allelic Replacement in Staphylococcus aureus. Appl Environ Microbiol 78:3846–3854.
2. **Linder P, Lemeille S, Redder P.** 2014. Transcriptome-Wide Analyses of 5′-Ends in RNase J Mutants of a Gram-Positive Pathogen Reveal a Role in RNA Maturation, Regulation and Degradation. PLoS Genet 10.
3. **Charpentier E, Anton AI, Barry P, Alfonso B, Fang Y, Novick RP.** 2004. Novel Cassette-Based Shuttle Vector System for Gram-Positive Bacteria. Appl Environ Microbiol 70:6076–6085.
