# Supplementary material for: An Essential Factor for High Mg2+ Tolerance of Staphylococcus aureus
Source: Front Microbiol. 2016 Nov 25;7:1888. doi: 10.3389/fmicb.2016.01888 (PMC5122736; doi:10.3389/fmicb.2016.01888)
Supplement: Supplementary file 2 [file Image_1.PDF]

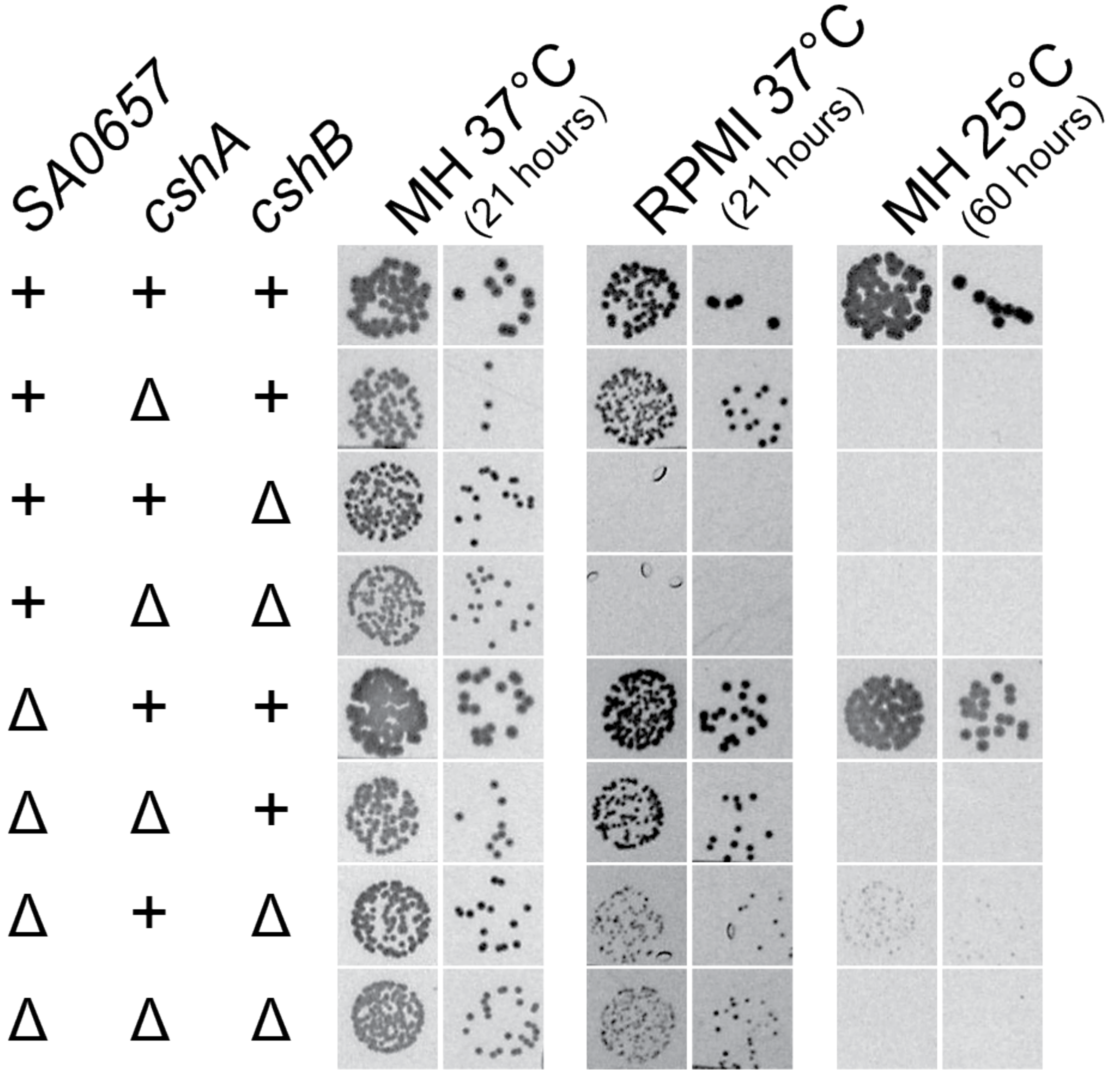


**Figure S1, Reconstruction of *ΔSA0657* in clean backgrounds confirms the suppression of *ΔcshB*, and that this suppression is independent of CshA presence.**

Two dilutions of each strain were spotted on either MH- or RPMI-agar, and incubated at the indicated temperatures and times. A + signifies the wild-type allele and a Δ indicates a chromosomal deletion of the gene. The reconstructed deletion of *ΔSA0657* suppresses the RPMI phenotype of *ΔcshB*, and a slight suppression of the cold-sensitivity can also be observed when comparing the growth at 25°C between the *ΔcshB* and *ΔcshBΔSA0657* strains.
