# Supplementary material for: An Essential Factor for High Mg2+ Tolerance of Staphylococcus aureus
Source: Front Microbiol. 2016 Nov 25;7:1888. doi: 10.3389/fmicb.2016.01888 (PMC5122736; doi:10.3389/fmicb.2016.01888)
Supplement: Supplementary file 3 [file Image_2.PDF]

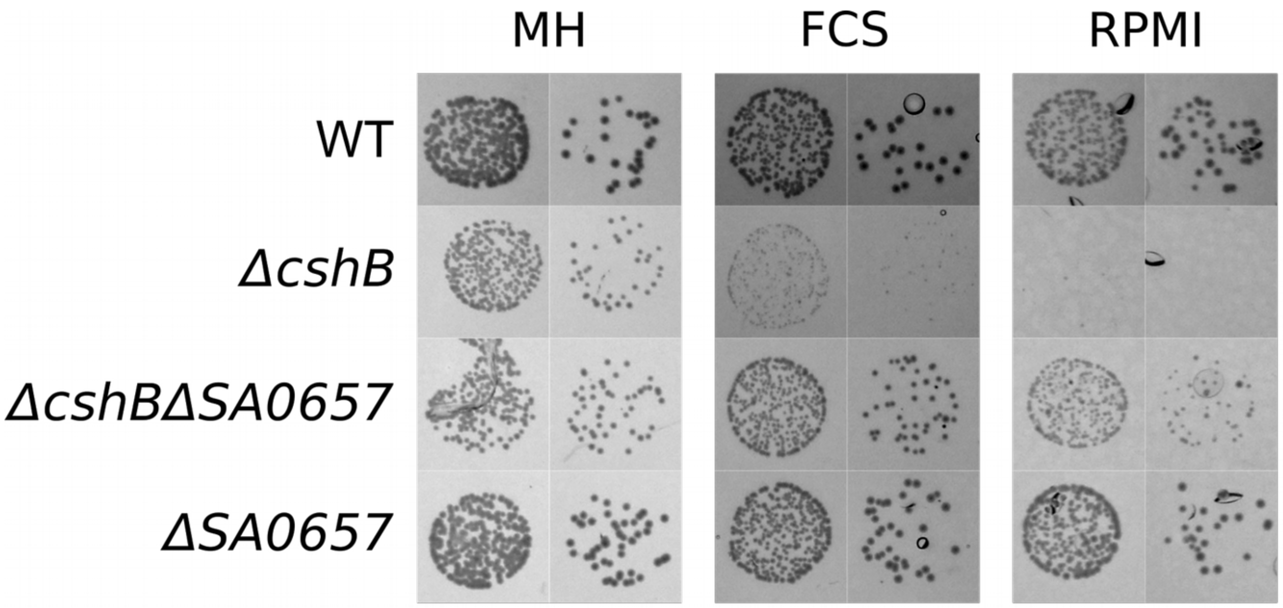

Figure S2, *ΔSA0657* suppresses *ΔcshB slow growth* in serum and RPMI. Two dilutions from overnight cultures of each strain were spotted on MH-, RPMI- or FCS (Fetal Calf Serum)-agar, and incubated at the indicated temperatures and times. The *ΔcshB* strain grows poorly on FCS and its synthetic substitute RPMI, while growth can be restored by deletion of *SA0657*. The upper part of the figure is from Figure 1A.
