# Supplementary material for: An Essential Factor for High Mg2+ Tolerance of Staphylococcus aureus
Source: Front Microbiol. 2016 Nov 25;7:1888. doi: 10.3389/fmicb.2016.01888 (PMC5122736; doi:10.3389/fmicb.2016.01888)
Supplement: Supplementary file 4 [file Image_3.PDF]

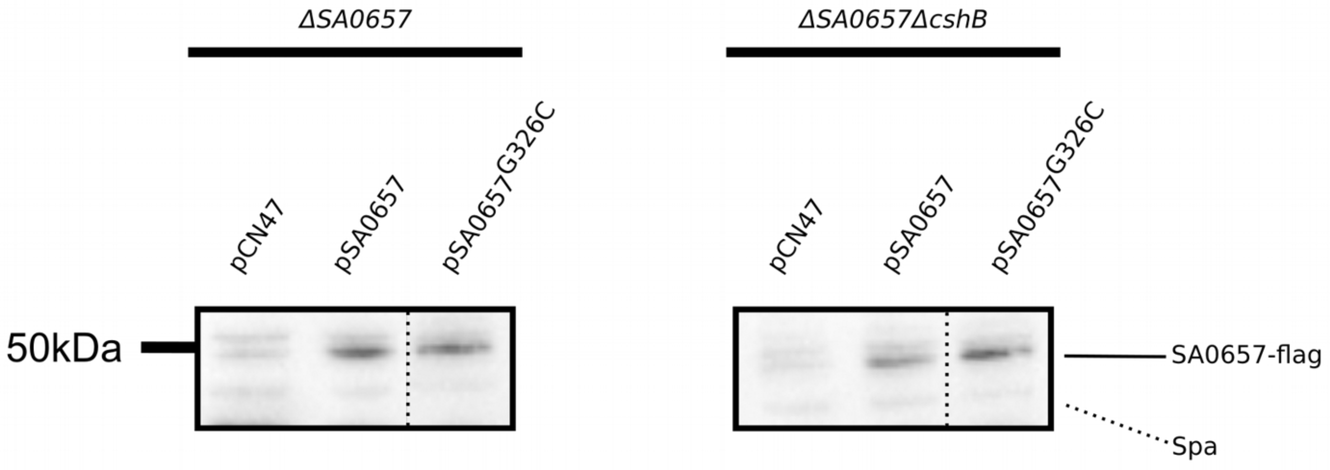

Figure S3, Western blot anti-flag-SA0657. The arrow indicates the band at the expected size for SA0657-flag (~52kDa). Protein A (Spa) band is indicated by a dotted arrow. All six lanes presented here were cropped from the same membrane.

Material and methods: Overnight cultures were diluted to OD_{600}=0.05 and grow in MH medium supplemented with erythromycin to exponential phase (OD_{600}=0.4) in shaking conditions at 37°C. 10 mL of culture were harvested by centrifugation (5000 rpm for 10 min) and subsequently lysed in PBS buffer containing 0.2 mg/ml lysostaphin, 1 unit of DNase I and complete anti-protease (Roche) at 37°C for 15 minutes. Protein amounts were quantified by Bradford. 5 µg of protein were migrated on 12% acrylamide gel (Life technologies) and then transferred to PVDF membrane. Proteins of interest were revealed using anti-flag (Sigma F3165) at 1/5000 and secondary anti-mouse antibody at 1/5000. Revelation was performed with HCL prime reagents (GE Healthcare, Glattbrugg, Switzerland)
