# Supplementary material for: An Essential Factor for High Mg2+ Tolerance of Staphylococcus aureus
Source: Front Microbiol. 2016 Nov 25;7:1888. doi: 10.3389/fmicb.2016.01888 (PMC5122736; doi:10.3389/fmicb.2016.01888)
Supplement: Supplementary file 5 [file Image_4.PDF]

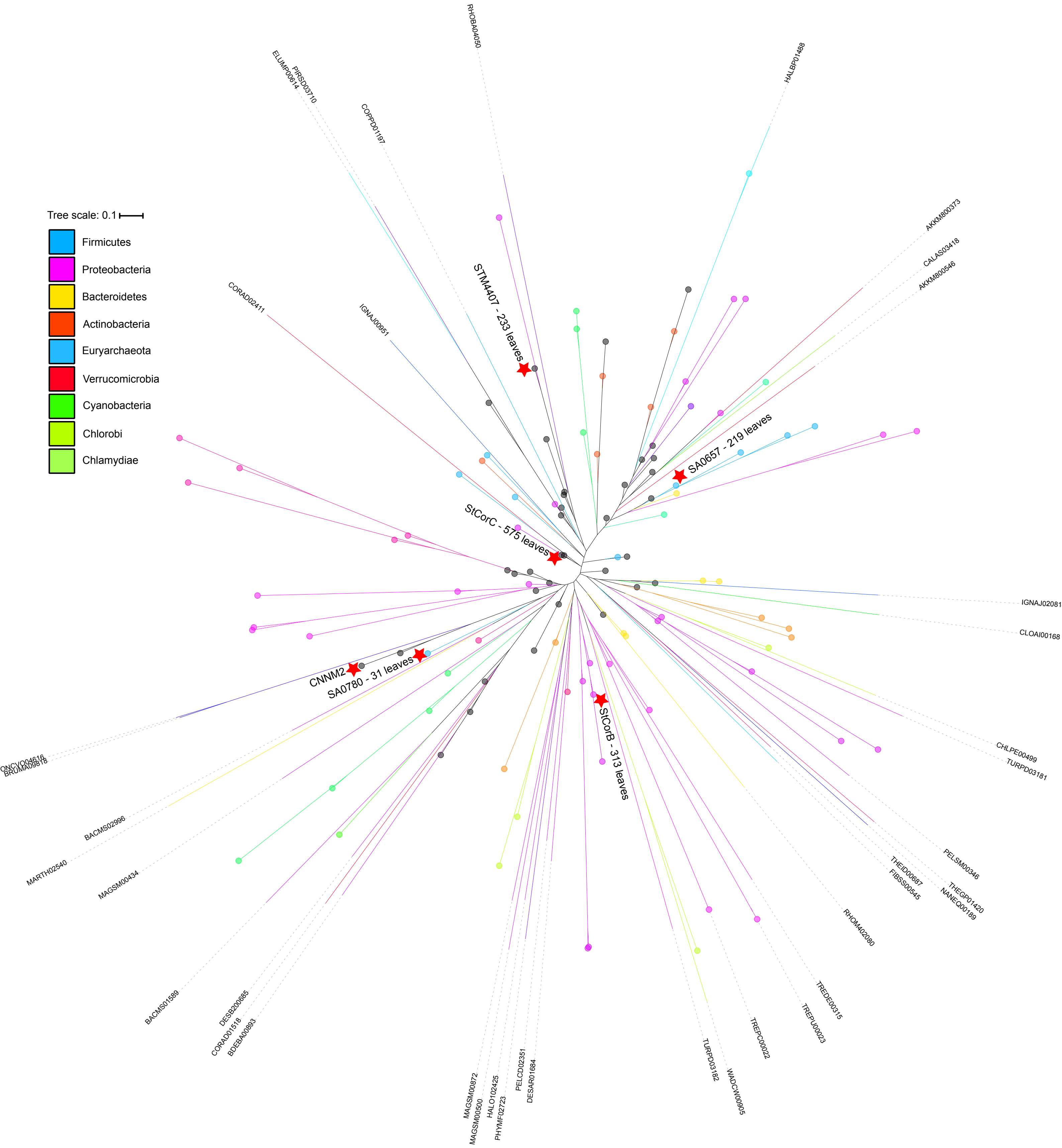

Tree scale: 0.1
Firmicutes
Proteobacteria
Bacteroidetes
Actinobacteria
Euryarchaeota
Verrucomicrobia
Cyanobacteria
Chlorobi
Chlamydiae
STM4407 - 233 leaves
SA0657 - 219 leaves
StCorC - 575 leaves
CNNM2
SA0780 - 31 leaves
StCorB - 313 leaves
RHOBA04050
HALBP01488
PIRSD03710
ELUMP00614
COPPD01197
CORAD02411
IGNAJ00951
AKKM800373
CALAS03418
AKKM800546
IGNAJ02081
CLOAI00168
CHLPE00499
TURPD03181
PELSM00346
THEID00687
FIBSS00545
THEGP01420
NANEQ00189
RHOM402080
TREDE00315
TREPU00023
TREPC00022
TURPD03182
WADCW00905
MAGSM00872
MAGSM00500
HALO102425
PHYMF02723
PELCD02351
DESAR01684
BACMS01589
DESB200685
CORAD01518
BDEBA00893
BACMS02996
MARTH02540
MAGSM00434
ONCVO04616
BRUMA09818

Figure S4: Phylogenetic tree of CorB/MrfA orthologs. SA0657, SA0780, STM2679, STM4407, human and murin CNNM2 orthologs according to the OMA database were aligned by clustal and the tree was rendered by Itol (http://itol.embl.de/). All clades with branch length inferior to 0.6 were collapsed. Color reflect phylum. A black collapsed node indicates several phyla are represented. Proteins mentioned in the manuscript are indicated by a star. An interactive version of this tree is available at http://itol.embl.de/tree/129194873136171465907480
