# Supplementary material for: An Essential Factor for High Mg2+ Tolerance of Staphylococcus aureus
Source: Front Microbiol. 2016 Nov 25;7:1888. doi: 10.3389/fmicb.2016.01888 (PMC5122736; doi:10.3389/fmicb.2016.01888)
Supplement: Supplementary file 6 [file Image_5.PDF]

```
AF085347      caccaccacgctgatgatcatattaatcatcatggtggtcatctcggcctactatttttc
STM2679       ----------------------------------ATGGTGGTCATCTCGGCCTATTTTTC
                                                 ****  **    *   *********

AF085347      cggttccgaaactggaatgatgacgctaaaccgttaccgtttacgccatatggccaagca
STM2679       CGGTTCCGAAACTGGAATGATGACGCTAAACCGTTACCGTTTACGCCATATGGCGAAGCA
              ****************************************************** *****

AF085347      aggtaatcgctcggcaaaacgggtagaaaaattattgcgtaaacccgatcgactgataag
STM2679       AGGTAATCGCTCGGCAAAACGGGTAGAAAAATTATTGCGTAAACCCGATCGACTGATAAG
              ************************************************************

AF085347      cctggtgctgatcggcaataacctggtcaacattcttgcctccgcgctcggcacgattgt
STM2679       CCTGGTGCTGATCGGCAATAACCTGGTCAACATTCTTGCCTCGGCGCTCGGCACGATTGT
              ***************************************** ******************

AF085347      gggtatgcggttgtacggcgacgccggcctggctatcgccaccggggtactgacgtttgt
STM2679       GGGTATGCGGCTTTACGGCGACGCCGGCGTGGCTATCGCCACCGGGGTACTGACGTTTGT
              ********** * *************** *******************************

AF085347      cgtgttagtttttgctgaagtcctgcccaaaaccatcgccgcgctctatccggaaaaagt
STM2679       CGTGTTAGTTTTTGCTGAAGTCCTGCCCAAAACTATCGCCGCGCTCTATCCGGAAAAAGT
              ******************************** ***************************

AF085347      ggcctatcccagcagcttcctgctggcgccgttacagatactgatgatgccgctggtgtg
STM2679       GGCCTATCCCAGCAGCTTCCTGCTGGCGCCGTTACAGATACTGATGATGCCGCTGGTGTG
              ************************************************************

AF085347      gttacttaacaccatcacccgcctgctgatgcgtatgatgggaattaaaacggacatcgt
STM2679       GTTACTTAACACCATCACCCGCCTGCTGATGCGTCTGATGGGAATTAAAACGGACATCGT
              ********************************** *************************

AF085347      ggtcagcgggtcgttaagcaaagagcaattacgcactatcgtgcatgaatcccgttcgca
STM2679       GGTCAGCGGGTCGTTAAGCAAAGAGGAATTACGCACTATCGTGCATGAATCCCGTTCGCA
              ************************* **********************************

AF085347      aatctctcgccgcaatcaggatatgctgctgtcggttctcgatcctggaaaagatgaccg
STM2679       AATCTCTCGCCGCAATCAGGATATGCTGCTGTCGGTTCTCGATC-TGGAAAAGGTCAGCG
              ******************************************** ******** * * **

AF085347      ttgatgacatcatggtgccgcgtaatgaaattatcgggatcgatatcaatgacgactgga
STM2679       TGGATGACATCATGGTGCCGCGTAATGAAATTATCGGGATCGATATCAATGACGACTGGA
              * **********************************************************

AF085347      agtctatcgagcggcagctcacccactcgccgcacggacgcattgtgctctatcgcgatt
STM2679       AGTCTATCGAGCGGCAGCTCACCCACTCGCCGCACGGACGCATTGTGCTCTATCGCGATT
              ************************************************************

AF085347      cgctggatgacgccatcagtatgctgcgcgtgcgtgaagcctggcggttaatggccgaga
STM2679       CGCTGGATGACGCCATCAGTATGCTGCGCGTGCGTGAAGCCTGGCGGTTAATGGCCGAGA
              ************************************************************

AF085347      aaaaagagttcaccaaagagatgatgctgcgcgccgccgatgaaatctattacgtcccgg
STM2679       AAAAAGAGTTCACCAAAGAGATGATGCTGCGCGCCGCCGATGAAATCTATTACGTCCCGG
              ************************************************************

AF085347      aaggtacgccgctcagtacgcaactgataaaatttcagcgcaataaaaagaaagtcggac
STM2679       AAGGTACGCCGCTCAGTACGCAACTGATTAAATTTCAGCGCAATAAAAAGAAAGTCGGAC
              **************************** *******************************

AF085347      tggtcgtcaacgaatatggcgatatccaggggctggtcaccgtggaagatattctcgagg
STM2679       TGGTCGTCAACGAATATGGCGATATCCAGGGGCTGGTCACCGTGGAAGATATTCTCGAGG
              ************************************************************

AF085347      agatcgttggcgacttcaccacatcgatgtcgccgacactggcggaagaggtcacgccgc
STM2679       AGATCGTTGGCGACTTCACCACATCGATGTCGCCGACACTGGCGGAAGAGGTCACGCCGC
              ************************************************************

AF085347      aaaacgacggttcggtcattattgacggtaccgccaacgtccgggaaatcaataaagcgt
```

```
STM2679            AAAACGACGGTTCGGTCATTATTGACGGTACCGCCAACGTCCGGGAAATCAATAAAGCGT
                   ************************************************************

AF085347           ttaactggcacctgccggaagatgacgcgcgcaccgtcaacggggtgattctggaggcgc
STM2679            TTAACTGGCACCTGCCGGAAGATGACGCGCGCACCGTCAACGGGGTGATTCTGGAGGCGC
                   ************************************************************

AF085347           tggaggaaattccggttgctggcacgcgcgtgcgcattgagcagtacgatatagatattc
STM2679            TGGAGGAAATTCCGGTTGCTGGCACGCGCGTGCGCATTGAGCAGTACGATATAGATATTC
                   ************************************************************

AF085347           tcgatgtacaggaaaatatgattaaccaggtaaaggttgtaccggtaaaaccgctgcgcg
STM2679            TCGATGTACAGGAAAATATGATTAAGCAGGTAAAGGTTGTACCGGTAAAACCGCTGCGCG
                   ************************* **********************************

AF085347           agagtgtggcggagtaacacaatggcgaactccggttcgcctttttatcgccgtctttt
STM2679            AGAGTGTGGCGGAGTAA-------------------------------------------
```

```
AF085347(CorF)     MIILIIMVVISAYYFSGSETGMMTLNRYRLRHMAKQGNRSAKRVEKLLRKPDRLISLVLI
AF085347(CorB)     ------------------------------------------------------------
STM2679            -------MVVISAYFSGSETGMMTLNRYRLRHMAKQGNRSAKRVEKLLRKPDRLISLVLI

AF085347(CorF)     GNNLVNILASALGTIVGMRLYGDAGLAIATGVLTFVVLVFAEVLPKTIAALYPEKVAYPS
AF085347(CorB)     ------------------------------------------------------------
STM2679            GNNLVNILASALGTIVGMRLYGDAGVAIATGVLTFVVLVFAEVLPKTIAALYPEKVAYPS

AF085347(CorF)     SFLLAPLQILMMPLVWLLNTITRLLMRMMGIKTDIVVSGSLSKEQLRTIVHESRSQISRR
AF085347(CorB)     ------------------------------------------------------------
STM2679            SFLLAPLQILMMPLVWLLNTITRLLMRLMGIKTDIVVSGSLSKEELRTIVHESRSQISRR

AF085347(CorF)     NQDMLLSVLDPGKDDR--------------------------------------------
AF085347(CorB)     -------------MTVDDIMVPRNEIIGIDINDDWKSIERQLTHSPHGRIVLYRDSLDDA
STM2679            NQDMLLSVLDLEKVSVDDIMVPRNEIIGIDINDDWKSIERQLTHSPHGRIVLYRDSLDDA

AF085347(CorF)     ------------------------------------------------------------
AF085347(CorB)     ISMLRVREAWRLMAEKKEFTKEMMLRAADEIYYVPEGTPLSTQLIKFQRNKKKVGLVVNE
STM2679            ISMLRVREAWRLMAEKKEFTKEMMLRAADEIYYVPEGTPLSTQLIKFQRNKKKVGLVVNE

AF085347(CorF)     ------------------------------------------------------------
AF085347(CorB)     YGDIQGLVTVEDILEEIVGDFTTSMSPTLAEEVTPQNDGSVIIDGTANVREINKAFNWHL
STM2679            YGDIQGLVTVEDILEEIVGDFTTSMSPTLAEEVTPQNDGSVIIDGTANVREINKAFNWHL

AF085347(CorF)     ------------------------------------------------------------
AF085347(CorB)     PEDDARTVNGVILEALEEIPVAGTRVRIEQYDIDILDVQENMINQVKVVPVKPLRESVAE
STM2679            PEDDARTVNGVILEALEEIPVAGTRVRIEQYDIDILDVQENMIKQVKVVPVKPLRESVAE
```

Figure S5, Alignment of the original and partial sequences of *StcorB* and *STM2679*.

Original sequence for *StcorB* (AF130857) locus was obtained from partial sequencing. Sequencing errors in this original data suggested *StcorB,* coding for a predicted cytosolic protein, was preceded by a short locus termed *corF,* predicted to be an integral membrane protein. Full genome sequencing reveals *StcorB* (STM2679) is an ORF of 1239 nucleotides encompassing both original ORFs. Alignment of the sequences by clustalΩ shows the location of the errors in the original sequencing.
