# Supplementary figures and images for: An Essential Factor for High Mg2+ Tolerance of Staphylococcus aureus
Source: Front Microbiol. 2016 Nov 25;7:1888. doi: 10.3389/fmicb.2016.01888 (PMC5122736; doi:10.3389/fmicb.2016.01888)

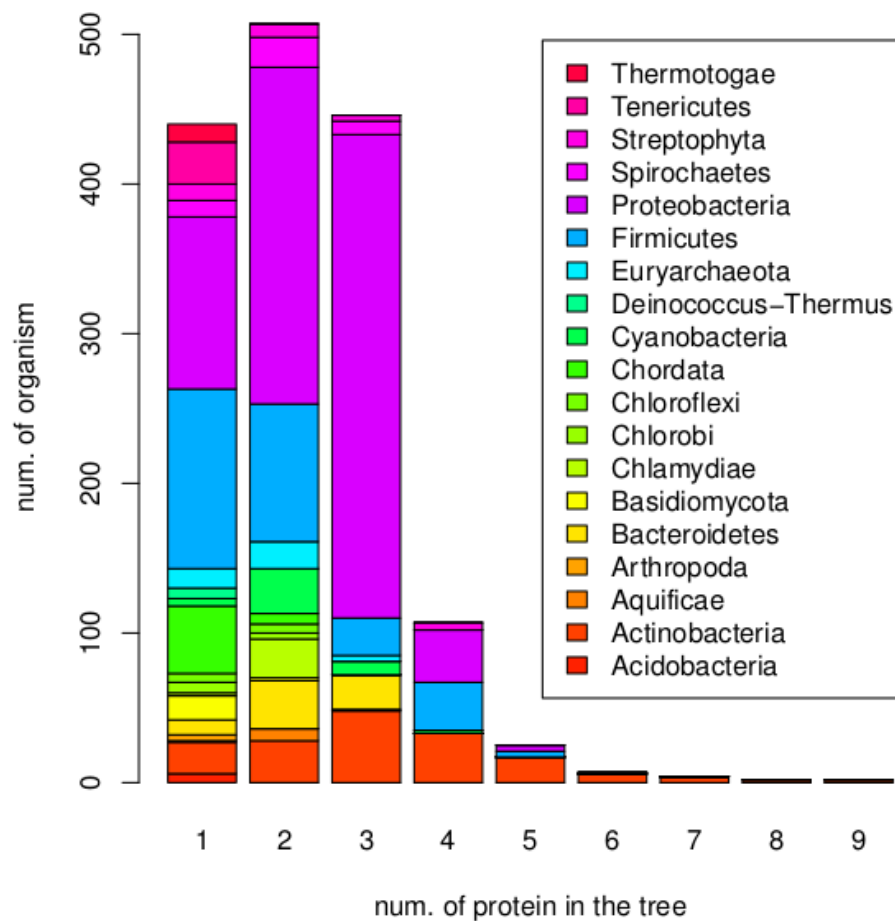

**Figure S6, Barplot representing the number of CorB-like protein genes per organism.**

Supplement: Supplementary file 7 [file Image_6.PDF]
