# Supplementary material for: An Essential Factor for High Mg2+ Tolerance of Staphylococcus aureus
Source: Front Microbiol. 2016 Nov 25;7:1888. doi: 10.3389/fmicb.2016.01888 (PMC5122736; doi:10.3389/fmicb.2016.01888)
Supplement: Supplementary file 8 [file Image_7.PDF]

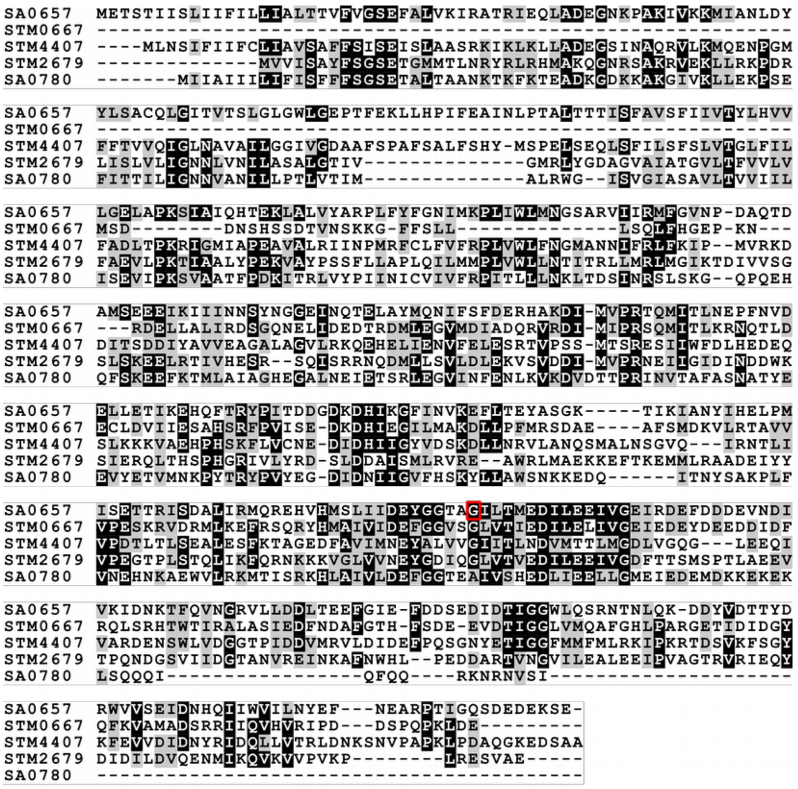

	SA0657	STM0667	STM4407	STM2679	SA0780
SA0657	100.00				
STM0667	27.24	100.00			
STM4407	25.46	22.46	100.00		
STM2679	22.03	23.93	22.47	100.00	
SA0780	23.68	19.91	23.01	27.54	100.00

Figure S7, Alignment of SA0657, SA0780, STM0667 (StCorC), STM2679 (StCorB) and STM4407 (StCorB paralog)

The alignment and percentage identity matrix were calculated using clustalΩ (1.2.1). Gly326 residue is highlighted by a red square.
