# Supplementary material for: An Essential Factor for High Mg2+ Tolerance of Staphylococcus aureus
Source: Front Microbiol. 2016 Nov 25;7:1888. doi: 10.3389/fmicb.2016.01888 (PMC5122736; doi:10.3389/fmicb.2016.01888)
Supplement: Supplementary file 9 [file Image_8.PDF]

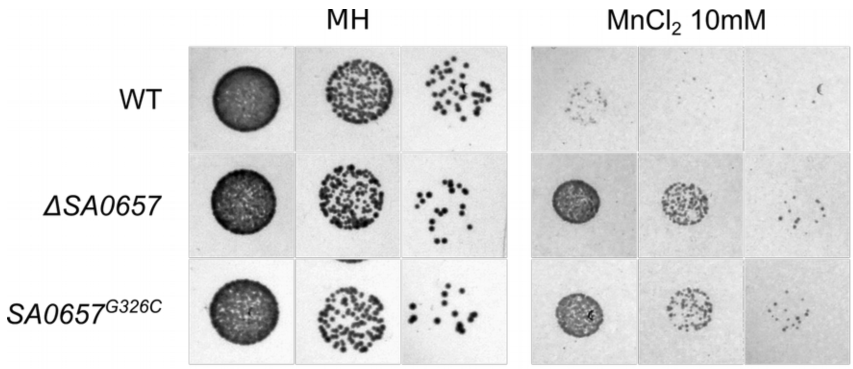

.

Figure S8, SA0657 mutants are more resistant to Mn^{2+}. MH-agar plates were complemented with 10mM $MnCl_2$, or not.
