# Supplementary material for: An Essential Factor for High Mg2+ Tolerance of Staphylococcus aureus
Source: Front Microbiol. 2016 Nov 25;7:1888. doi: 10.3389/fmicb.2016.01888 (PMC5122736; doi:10.3389/fmicb.2016.01888)
Supplement: Supplementary file 10 [file Image_9.PDF]

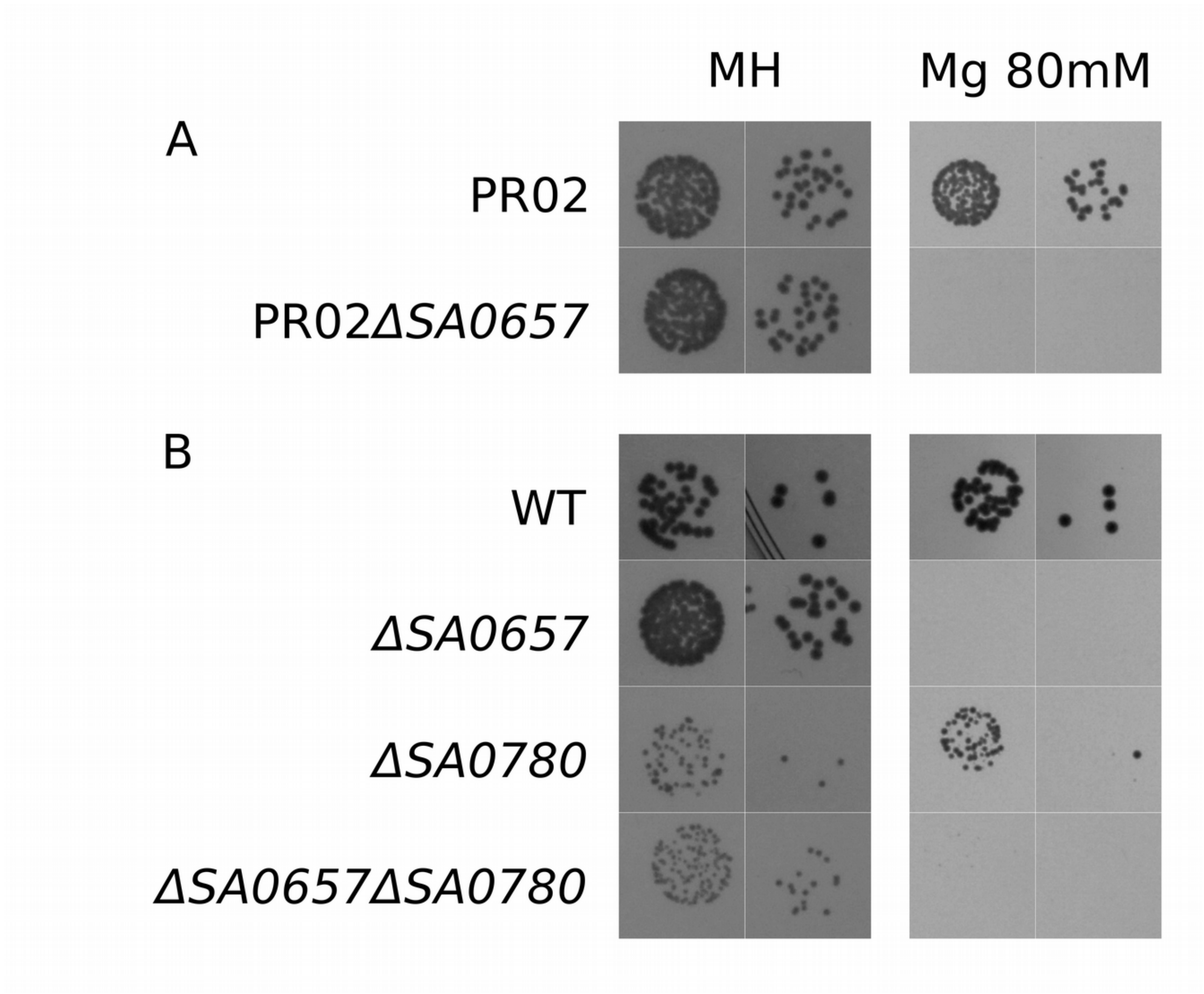

Figure S9, Growth of SA0657 and SA0780 mutants in presence of magnesium.

MH-agar plates were complemented with 80mM $MgCl_2$ or not, and grown for 24h at 37°C. A, deletion of SA0657 renders PR02 (RN4220Δ*pyrEF*) Mg^{2+} sensitive. B, Growth of an SA0780 deletion mutant is unaffected by Mg^{2+} concentrations that inhibit the SA0657.
